# Current Strategies for Real-Time Enzyme Activation

**DOI:** 10.3390/biom12050599

**Published:** 2022-04-19

**Authors:** Fang Wang, Yuchen Liu, Chang Du, Renjun Gao

**Affiliations:** Key Laboratory for Molecular Enzymology and Engineering, The Ministry of Education, School of Life Science, Jilin University, Changchun 130021, China; wangfang261621@163.com (F.W.); liuyc1919@outlook.com (Y.L.); duchang199808@163.com (C.D.)

**Keywords:** activation strategies, enzyme activity, near infrared, alternating magnetic field, microwave, ultrasound irradiation

## Abstract

Enzyme activation is a powerful means of achieving biotransformation function, aiming to intensify the reaction processes with a higher yield of product in a short time, and can be exploited for diverse applications. However, conventional activation strategies such as genetic engineering and chemical modification are generally irreversible for enzyme activity, and they also have many limitations, including complex processes and unpredictable results. Recently, near-infrared (NIR), alternating magnetic field (AMF), microwave and ultrasound irradiation, as real-time and precise activation strategies for enzyme analysis, can address many limitations due to their deep penetrability, sustainability, low invasiveness, and sustainability and have been applied in many fields, such as biomedical and industrial applications and chemical synthesis. These spatiotemporal and controllable activation strategies can transfer light, electromagnetic, or ultrasound energy to enzymes, leading to favorable conformational changes and improving the thermal stability, stereoselectivity, and kinetics of enzymes. Furthermore, the different mechanisms of activation strategies have determined the type of applicable enzymes and manipulated protocol designs that either immobilize enzymes on nanomaterials responsive to light or magnetic fields or directly influence enzymatic properties. To employ these effects to finely and efficiently activate enzyme activity, the physicochemical features of nanomaterials and parameters, including the frequency and intensity of activation methods, must be optimized. Therefore, this review offers a comprehensive overview related to emerging technologies for achieving real-time enzyme activation and summarizes their characteristics and advanced applications.

## 1. Introduction

Enzymes, as natural biocatalysts, are widely used in chemical synthesis, biosensors, biopharmaceuticals, and genetic engineering owing to their excellent specificity, selectivity, and high efficiency [1,2,3]. However, the activity of natural enzymes is easily affected by the temperature, pH, and the surrounding environment because of their poor stability, difficult separation from the reaction medium, and activity inhibition, leading to the limitation of many practical applications. Hence, the precise enhancement of protein activity is important to understand complex biological signaling networks and to control biological function. Many efforts have been devoted to improving enzyme activity by means of various methods that were classified into conventional and real-time strategies to make enzymes exert a better potency.

Conventional methods of improving enzyme activity mainly include designing enzyme variants, chemical modifications, immobilization strategies, or relying on changes in the temperature, pH, and ionic strength. However, the enzyme activity is not increased precisely, and the process of changing enzymatic characteristics by using these conventional strategies is generally tedious and unfocused, while also requiring massive amounts of reactants, long reaction times, and high catalyst loads. For instance, enzyme mutation can promote favorable changes in enzymatic properties to obtain high catalytic activity, thermostability and stereoselectivity through rational design, directed evolution, and semirational design [4]. However, the process of screening mutants is laborious and time-consuming and may lead to hazardous mutations [5]. Chemical modification refers to the direct binding of an enzyme to another molecule to control many biological systems. Nevertheless, the main problem of chemical modification is that it may be difficult to achieve fully controllable modification and requires customized modification for each enzyme [6]. Meanwhile, enzyme immobilization can lead to the loss of activity, leakage from the carrier, and mass transfer limitations [7,8,9]. At the same time, enzymes are unstable outside of an optimal temperature, pH, and ionic strength range. These drawbacks impede the long-term operational reaction of enzymes and cannot fulfill sustainable, real-time controllable biocatalysts.

**Figure 1:**
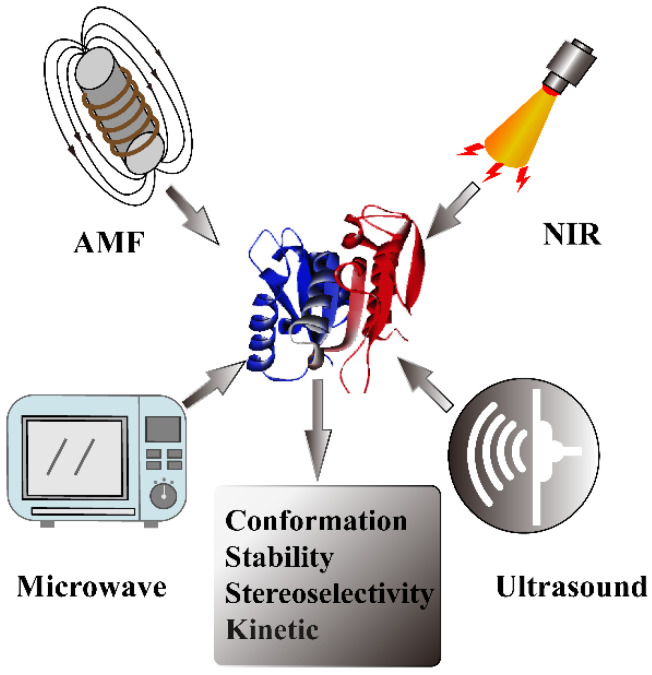
Strategies of real-time enzyme activation including near-infrared, microwave, ultrasound, and alternating magnetic field.

To overcome these disadvantages of conventional methods, many studies have paid close attention to exploring efficient activation means to achieve precise and rapid control of enzyme activity. Therefore, real-time activation includes near-infrared (NIR), alternating magnetic field (AMF), microwave, and ultrasound irradiation, which can make it available to remotely and spatiotemporally improve enzyme activity and maximize their biological function (Figure 1). Due to their sustainability, low invasiveness, and easy tunability, these technologies are highly appropriate for real-time controlling of enzymatic conformation, activity, and other properties and have emerged as an effective toolkit in various fields, such as cancer therapy, the food industry, and environmental engineering [10,11,12,13]. Furthermore, real-time activation strategies can also intensively improve the reaction rate, shorten the reaction time, and reduce the mass transfer resistance. According to the different mechanisms of real-time strategies, nanomaterials such as plasmonic nanoparticles that are responsive to NIR and magnetic nanoparticles stimulated by AMF have been widely applied for enzyme immobilization to transfer energy to control enzyme activity. Microwaves, ultrasound, and AMF can directly influence enzymatic behavior only for certain enzymes without the assistance of nanomaterials. However, few reviews have been published in recent years systematically elucidating and summarizing four different real-time activation strategies of enzymes. Thus, this review provides a deep understanding and accessible discussion associated with the mechanism of real-time activation of enzyme activity. Additionally, it is involved in activating factors influencing enzyme catalytic performance as well as their prospective applications.

## 2. Near-Infrared Strategy

### 2.1. Mechanism

Near-infrared (NIR) is considered to be an emerging strategy that enables accurate, remote, and noninvasive biotransformation to control enzyme activity. The universal NIR-activated strategy is the combination of enzymes and plasmonic nanoparticles by using immobilization methods such as cross-linking, physical adsorption, and encapsulation [14]. In the near-infrared (NIR) spectral regions, NIR-responsive nanomaterials exhibit prominent optical properties, including localized surface plasmon resonances (LSPRs) at the interface between nanoparticles and enzymes, which can convert optical energy to internal energy with high efficiency [15,16,17,18]. Therefore, the plasmonic effect of nanomaterials can make it available to achieve the remote and spatiotemporal activation for biocatalytic processes upon NIR irradiation.

Photothermal Effect: A few studies have been reported to demonstrate the mechanisms of the influence of plasmonic effects at the interface of nanoparticles on enzyme activity. First, the LSPRs of plasmonic nanoparticles can transform optical energy into thermal energy, which leads to elevated local temperature on the nanoparticle surface and enables them to act as nanoscale heating elements upon near-infrared irradiation. The photothermal effect is the prevalent explanation to illustrate the mechanisms involved in the enzyme activation [19,20,21,22,23,24,25]. The photothermal effect of plasmonic nanostructures is suitable to increase the activity of thermophilic enzymes, which are highly stable and active at high temperatures [20,25]. Our group found that light-driven heat on gold nanorods can activate the activity of immobilized thermophilic enzymes, notably at different laser powers (0.5–2 W) [20]. The results showed that the AuNP-enzyme nanobiocatalytic systems exhibit higher catalytic efficiency (8-fold) than the free enzyme. However, light-to-heat conversion can also threaten conventional enzyme conformations, thereby resulting in the deactivation of the enzymes or a decrease in activity [26,27,28]. To address this issue, extensive efforts have been devoted to developing appropriate conjunction strategies between conventional enzymes and plasmonic nanoparticles to overcome the limitation of photothermal effects. For example, a simple and highly efficient stabilization approach was designed for immobilizing enzymes on plasmonic nanoheaters and encapsulating them through an in situ-polymerized porous organosilica layer [21]. The encapsulation strategy not only improves the thermal stability and cycle of reuses but also enhances the catalytic activity with NIR irradiation. Moreover, metal–organic frameworks (MOFs) can also be exploited to prevent enzyme-AuNPs from thermal deactivation and decrease activity due to their high thermal stability [23].

**Figure 2:**
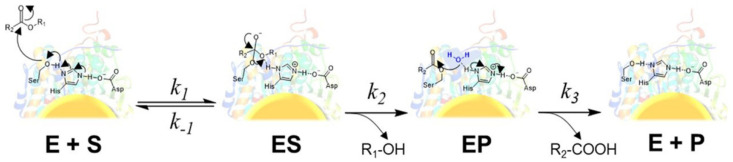
The mechanism of hydrolysis reaction catalyzed by a lipase. Reprinted with permission from de Barros et al., American Chemical Society [14].

Additionally, de Barros et al. proposed that the photothermal effect can change enzyme kinetics under external light irradiation. They used lipase (CALB) adsorbed on the surface of Au nanoparticles as a model system to explain the effect of LSPR excitation on the enzymatic activity by exploring the well-known three-step catalytic mechanism of hydrolases. The result showed that both k_cat_ and k_m_ of CALB-AuNPs catalyzed hydrolysis were improved after NIR irradiation, indicating that laser irradiation can affect the enzymatic activity due to the photothermal effect at the AuNPs surface [14]. To investigate which steps of enzyme catalysis are affected by light, including chemical hydrolysis, product release, or both steps, reaction-time courses were monitored under light conditions (Figure 2). According to the experiment, light increased the k_3_ of AuNSt@CALB twofold by favoring product release from the enzyme and hardly affected the kinetics of water attack of the hydrolysis step (k_1_, k_2_). The promotion of enzyme kinetics confirmed that photothermal heating of AuNPs remarkably changed the rate-limiting step of the enzymatic reaction by accelerating the product release.

Therefore, the enzyme activity can be improved by NIR-induced photothermal effects, which are remarkably beneficial for many biocatalytic processes. Furthermore, mechanistic studies of electron transport between nanoparticles (CdS) and enzymes induced by light have been carried out, which arguably results in enzymatic activity changes [18]. These studies revealed that the mechanism of light-activated enzyme activity is, to a large extent, dependent on the category of enzyme and the properties of nanoparticles. Therefore, a thorough comprehension of the interaction between the nanoparticle and enzyme is beneficial for the construction of enzyme-nanomaterial biocatalysts. This activation strategy, through combining enzymes with plasmonic nanomaterials stimulated by NIR, potentially revealed that there was no limitation for the type of enzyme. The high photothermal effect can quickly activate enzyme activity in a short time, but sometimes it may cause enzyme inactivation.

Light-activated photoenzymes: Besides the photothermal effect of plasmonic nanomaterials that activate enzymes, there are also a few natural photoenzymes, including protochlorophyllide oxidoreductase (POR), DNA photolyase, and fatty acid photodecarboxylase (FAP) that can be directly activated by light when catalyzing biochemical processes [29]. The specificity of these photoenzymes contains cofactors or groups with light-harvesting properties, which can capture light energy to accelerate biocatalytic reactions. For example, the cofactor flavin adenine dinucleotide (FAD) of DNA photolyase absorbs light to initiate cleavage of the two major DNA photodamages such as cyclobutane pyrimidine dimers and photoproducts [30]. As the cofactor of FAP, FAD, with its high light-absorbing properties, can promote the electron transfer from the substrate fatty acids to FAD, which assists the decarboxylation of free fatty acids to n-alkanes or -alkenes using light [31]. Furthermore, protochlorophyllide oxidoreductase can capture the excitation energy to drive the subsequent hydride- and proton-transfer in the reduction of the C17–C18 double bond of protochlorophyllide (Pchlide) to form chlorophyllide [32]. Therefore, the light-activated photoenzymes can effectively improve their biotransformation rate under light, and the photoexcited cofactor can be applied to create an enzyme with light-harvesting properties [33].

### 2.2. NIR-Responsive Nanomaterials

**Figure 3:**
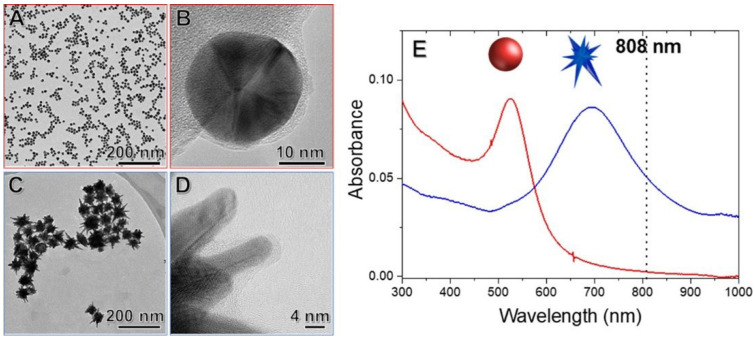
(**A**,**B**) TEM images of AuNSp@CALB; (**C**,**D**) TEM images of AuNSt@CALB; (**E**) UV−vis spectra of AuNSp@CALB (red line) and AuNSt@CALB (blue line). Reprinted with permission from de Barros et al., American Chemical Society [14].

Gold nanoparticles: The development of NIR-responsive nanoparticles plays an important role in enzyme activity activated by near-infrared light through their excellent optical properties (Table 1). Gold nanoparticles are the most frequently used plasmonic nanomaterial due to their light absorption, efficient photothermal conversion, high biocompatibility, and easy surface modification [34,35]. The localized surface plasmon resonances of AuNPs are intensively influenced by the dimensions, morphology, and surrounding environment [36], thereby affecting the catalytic activity of immobilized enzymes under NIR irradiation. NP size, which directly correlates with surface area and curvature, probably influences enzyme attachment on NPs. Joyce et al. investigated the effect of NP size on the catalytic activity of phosphotriesterase (PTE) by employing a series of AuNPs with increasing size [37]. The results showed that the smaller-sized AuNPs (10 nm) exhibit the highest catalytic efficiency, which improves PTE kcat by up to 10-fold, while the 100 nm NPs only increase PTE kcat by four-fold. This is because the rate-limiting step of enzyme−product release can be affected by AuNP size and curvature. Furthermore, the 10 nm AuNPs attained the highest conjunction efficiency with PTE at 97% and achieved a higher initial rate in the reaction of substrate paraoxon conversion. Additionally, the morphology of gold nanoparticles, including gold nanorods, gold nanospheres (AuNSp), and nanostars (AuNSt), is also very significant because of their optoelectronic and physicochemical properties [38,39,40]. Gold nanorods (AuNRs), with sophisticated shapes and great optical plasmon properties, are likely to be the most prevalent nanomaterials for enzyme immobilization, and they have been used in disease therapy and biosynthesis [41]. Moreover, de Barros et al. immobilized lipase on Au nanospheres and nanostars to compare the effect of NIR excitation on the hydrolytic activity of AuNSt@CALB and AuNSp@CALB (Figure 3) [14]. It was found that AuNSt@CALB achieved higher enzyme activity than AuNSp@CALB. This is because AuNSt has better matched LSPR positions that can significantly respond to different light excitation wavelengths. Hence, these studies suggest that both the size and shape of nanoparticles are important elements to determine the responsiveness to NIR irradiation.

Other NIR-responsive nanomaterials: In addition to Au nanoparticles, other nanoparticles with photothermal effects, including platinum (Pt) nanoparticles and Ti3C2TX nanosheets, are also exploited as nanoheaters that can convert NIR light into heat [28,42,43]. Interestingly, Pt nanoparticles are usually inserted into the interior of the enzyme structure due to their ultrasmall size to control the off-on switching of enzyme activity. Zhang et al. fabricated a thermoresponsive enzyme-Pt-polymer biocatalytic system by embedding Pt nanoparticles inside an enzyme decorated with an amphiphilic copolymer of acrylamide and acrylonitrile [42]. The thermosensitive copolymer can become microscale aggregates at a temperature below the UCST to encapsulate the enzyme structure, thereby inhibiting the catalytic activity. Nevertheless, the local heat of Pt nanoparticles under NIR irradiation can enable soluble copolymer to release enzyme activity. This suggests that it is possible to achieve precise and real-time enzyme activation switching. Furthermore, some special nanoparticles, including the abovementioned CdS nanocrystals and graphene oxide (GO), can absorb photons with various energy levels (wavelengths) because of their band gap energy upon NIR irradiation [18,43,44]. Therefore, these nanoparticles can be involved in the electron transfer of redox enzymes to manipulate catalytic activity [45].

### 2.3. Applications

**Figure 4:**
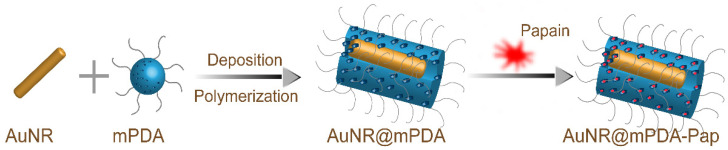
Schematic illustrations of preparation of mPDA encapsulated gold nanorods for papain loading.

Cancer therapy: The unique optical properties of plasmonic nanoparticles upon NIR irradiation have been widely applied for photothermal therapy and photodynamic therapy of cancer [46,47,48,49]. Many specific enzymes can selectively target the desired cells or degrade crucial biomacromolecules in tumors, making them prevalent as therapeutic agents for some diseases, which is called enzymatic therapy [50,51]. Therefore, the construction of enzyme-nanoparticle complexes is a promising platform that can contribute to multifunctional cancer therapy strategies by combining photothermal therapy and enzymatic therapy in one system [34,35]. However, the limited penetration depth of cancer therapy has been a major obstacle for many therapeutic strategies. In recent years, there has been growing interest in the second NIR (NIRII) window (1000–1350 nm) since it exhibits deeper tissue penetration, lower background signal, and higher maximum permission exposure than the traditional first NIR (NIR-I) window (650–950 nm) [52,53,54]. In addition, papain (pap), with superior proteolytic activity, good biocompatibility, and thermostability, can be used to deplete the tumor extracellular matrix (ECM), which is considered to be a physical barrier impeding tumor therapy. A recent study was devoted to establishing an NIR-II light-activated AuNR@mPDA-Pap nanosystem consisting of an AuNR core and a PEGylated mPDA shell for stromal depletion and deep-tumor therapy (Figure 4) [10]. AuNR@mPDA-Pap exhibited an excellent photothermal conversion efficiency of 56.5%, good photostability, and effective deep tissue ablation of up to 5 mm under NIR-II irradiation. The results also demonstrated that ECM digestion and tumor penetration are the result of the synergistic effect between the photothermal effect and enzymes because NIR-II treatment can penetrate deep tissue effectively and enhance the thermophilic enzymatic activity of degrading tumor stroma by the photothermal effect. Moreover, many studies have carried out enzyme-assisted photodynamic therapy (PDT) based on nanomaterials, which is also becoming an accepted therapeutic tool for cancer and many diseases [55,56,57,58].

**Figure 5:**
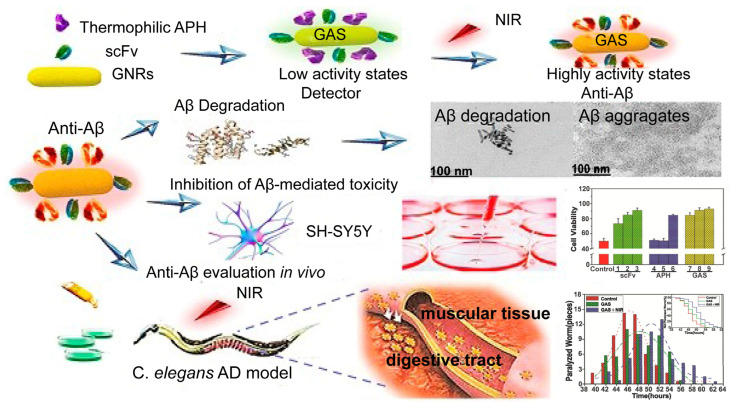
Inhibition of Aβ-mediated toxicity by GAS used for AD treatment. Reprinted with permission from Liu et al., Ivyspring International Publisher [41].

Neurodegenerative disease therapy: In addition to tumor therapy, enzyme-nanoparticle complexes show potential application for the therapy of neurodegenerative disease. Alzheimer’s disease, one of the most common neurodegenerative diseases, is caused by an abnormal accumulation of amyloid-β (Aβ) in different parts of the brain [59]. Therefore, great efforts have been made to develop theranostic strategies to degrade Aβ aggregates, including anti-Aβ aggregate agents, hyperthermia, and amyloid-degrading enzymes (ADEs) [60,61,62]. Based on this, our group designed a novel multifunctional anti-Aβ agent GNRs-APH-scFv (GAS) by combining AuNPs with an ADE (thermophilic APH ST0779) and modified it with a single-chain variable fragment (scFv12B4) that can target Aβ levels with high specificity (Figure 5) [41]. It is worth noting that the photothermal effect of the AuNPs upon NIR irradiation can activate not only thermozyme activity but also degrade the aggregates. Moreover, all the control groups, including APH and scFv alone, as well as the conjunction of APH and scFv, GNRs-APH, and GNRs-scFv, showed decreased degradation of Aβ fibril aggregation compared to GNRs-APH-scFv. This indicated that GAR-mediated hyperthermia and enzymatic therapy exhibited synergistic effects on dissociating Aβ aggregates and inhibiting Aβ-mediated toxicity. Thus, this GAR nanoplatform provided innovative prospects for biomedical theranostics for AD treatment.

Degradation and synthesis of organic compounds: Many important chemical reactions that can be applied for the degradation of organic pollutants and synthesis of chemical intermediates rely on highly chemo-, regio-, and stereoselective enzymes. Therefore, enzyme-nanomaterial composites activated by NIR are a potential way to route chemosynthetic pathways efficiently. Pan et al. constructed sporopollenin-exine-capsule (SEC) micromotors loaded with horseradish peroxidase (HRP) and modified with polydopamine (PDA) for the degradation of organic pollutants [63]. When exposed to NIR irradiation, HRP will be released from the hollow structure of SECs. As a result, the degradation efficiency for organic degradation of PDA-coated SEC micromotors under NIR irradiation was higher than that without NIR irradiation, accelerating the release of the HRP enzyme. Moreover, bubble generation with the trigger of NIR light enhanced the diffusion of the enzyme and increased the reaction between the enzyme and organic pollutant, thus improving the degradation efficiency. Li et al. used the NIR irradiation to activate enzyme-conjugated gold nanorod composites (EGCs) to catalyze the aldol reaction, which is one of the important reactions to form carbon-carbon bonds in organic chemistry [20]. Notably, the EGCs achieved higher catalytic efficiency and excellent conversion in a short time when exposed to NIR compared to the free enzyme. These studies showed the great potential of remote and real-time enzyme activation strategies that can be used for industrial applications.

## 3. Microwave Radiation Strategy

### 3.1. Mechanism

Thermal effect: Microwave radiation refers to electromagnetic waves with frequencies between 300 MHz and 300 GHz, and it easily clusters into bundles, is highly oriented, and linearly propagates. The mechanism of microwave heating is that microwaves can change the vibrational energy of many molecules with dielectric properties, which convert the energy of electromagnetic radiation into heat [64]. Compared to traditional heating, microwave heating exhibits several key features: (1) rapid heating because direct microwave irradiation heats instead of thermal conduction; (2) selective heating because of the different absorption and loss of microwave energy by diverse substances; and (3) the electromagnetic field effect [65].

Considering the aforementioned advantages, recent years have witnessed a rapid increase in the effect of microwave radiation on biological applications [66,67,68]. The main mechanism of enzyme activation under microwave radiation is that noncovalent interactions involving hydrogen bonding, hydrophobic bonding, and van der Waals forces that maintain a stable secondary structure of enzymes can be affected by electromagnetic energy. Zhang et al. [69] explored the influence of microwave radiation on the secondary structure of glucoamylase. The results showed that the composition of α-helices decreased gradually, while the composition of β-sheets, β-turns, and random coils increased under microwave radiation. This conformational change of the enzyme, generally from compact to flexible, remarkably influences enzymatic activity and properties, including thermal stability [70], selectivity [71], and kinetics [72]. Furthermore, microwave radiation can also affect the interaction between the enzyme active site and the substrate. Rokhati et al. [73] demonstrated that microwave-assisted cellulase exhibited higher V_max_ and lower K_m_ for hydrolyzing chitosan than using the shaker incubator, suggesting stronger binding between enzyme and substrate. The kinetic changes shortened the reaction time and improved the catalytic efficiency of cellulase under microwave irradiation. 

**Figure 6:**
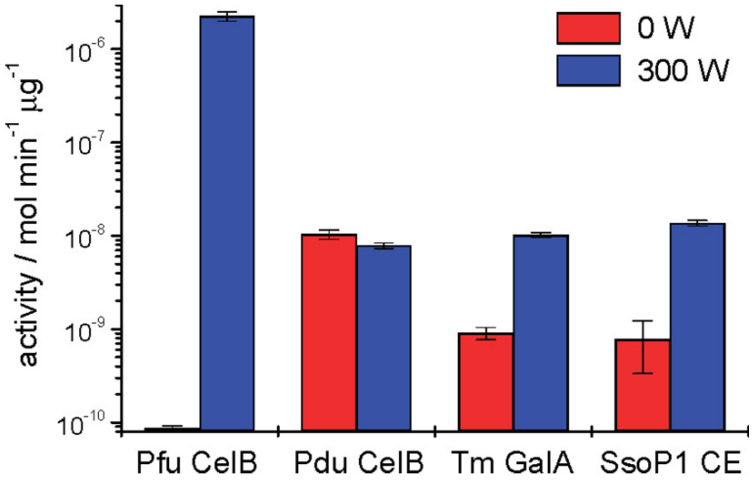
The Effect of microwave irradiation on different enzyme activity including Pfu CelB (β-glucosidase from *Pyrococcus furiosus*), Pdu CelB (β-glucosidase from *Prunus dulcis*), Tm GalA (α-galactosidase from *Thermotoga maritima*), and SsoP1 (carboxylesterase from *Sulfolobus solfataricus* P1). Reprinted with permission from Douglas et al., American Chemical Society [80].

Nonthermal effect: A few studies have been reported to demonstrate that some microwave-assisted reactions might be due to their nonthermal effect [74,75,76]. However, whether microwave radiation has an underlying nonthermal effect that can influence enzymatic reactions is under debate [77,78]. Nagashima et al. explored nonthermal microwave effects on β-glucosidase (optimal temperature: 60 °C) under 2.45 and 5.80 GHz microwave irradiation with an incubator to control the reaction temperature [79]. A 2.45 GHz microwave-treated enzyme exhibited maximal activity at 50 °C but became inactive at 60 °C. Moreover, the β-glucosidase exhibited a higher activity to catalyze the reaction within 20 min, while the convention heating required 30 min. Thus, 2.45 GHz microwaves not only decreased the optimum temperature but also improved the reaction rate, indicating the specific effect of microwaves compared to conventional heating. In contrast, 5.80 GHz microwaves did not make a difference in this reaction. They proposed a possible explanation at the molecular level that 2.45 GHz can affect water molecules and buffering ions, which might play an important role in the hydrolysis reaction by forming ion bonds with carboxyl groups on Glu or Asp of the enzyme, but 5.80 GHz only affected water molecules. Similarly, Young et al. also confirmed that β-glucosidase (CelB) from the hyperthermophilic archaeon (optimal temperature: 110 °C) could be activated at far below its optical temperature under 300 W microwave irradiation and that enzyme activity increased four-fold compared with the reaction without microwave radiation (Figure 6) [80]. However, a mesophilic homolog of CelB did not show any improvement under the same conditions. The potential mechanism was that the oscillating electric field could stimulate a dipole alignment of the hyperthermophilic peptide bonds, thereby promoting molecular motion. Although many reports have proposed the nonthermal effect of microwaves, whether the specific microwave effect exists is still ambiguous, and it seems to depend on the electric field frequency, power, temperature, or type of enzyme. In addition, it has been suggested that these studies lacked efficient experimental strategies, specific detection criteria, and reasonable discussion [81]. 

Overall, microwave irradiation normally generates efficient internal heating by directly transferring microwave energy to the enzyme, solvent, and reagent in the reaction mixture, and it can reduce the reaction time with a higher yield than conventional heating. However, almost all studies referring to the enzyme activation by microwave radiation are limited to several types of enzymes, including lipase, hydrolase, and glucosidase. Furthermore, even for the same type of enzyme, such as glucosidase, microwaves still have different or no activation effects on some enzymes from various sources [82]. Thus, many efforts should be devoted to further studying the mechanism of microwave-activated enzymes to advance the application of microwaves in various enzyme reactions.

### 3.2. Applications

**Figure 7:**
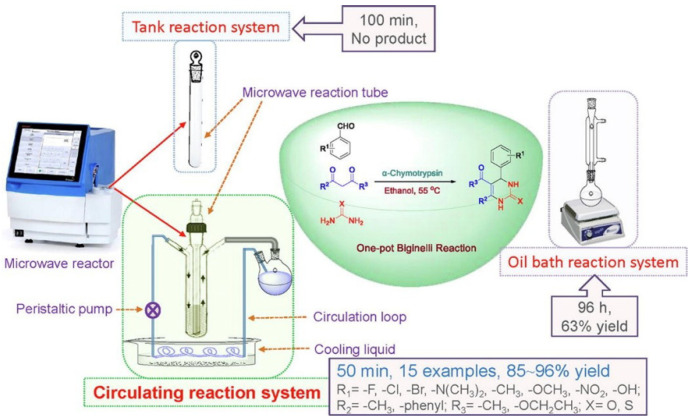
The structure of microwave reactor consists of a microwave reaction unit, cooling system unit, and circulating pump unit, which can enhance the effect of one-pot Biginelli reaction. All the reactions catalyzed by α-Chymotrypsin under microwave achieved 85–96% yield within 50 min. The conventional oil bath reaction system only obtained 63% yield within 96 h. Reprinted with permission from Xie et al., Elsevier [87].

Chemical industry: Microwave radiation, as a clean and green energy source, has attracted increasing attention in organic syntheses, such as peptide synthesis and drug synthesis [83]. Many researchers have shown interest in enzymatic reactions combined with microwaves to improve chemical synthesis [84,85,86]. Cases of α-chymotrypsin-catalyzed one-pot Biginelli reactions under microwave radiation in which the useful products 3,4-dihydropyrimidin-2-(1H)-ones (DHPMs) have been reported (Figure 7) [87]. DHPMs can be used as significant drug intermediates, including antioxidants, antiepileptics, and calcium channel antagonists [88,89]. After microwave radiation, α-chymotrypsin attained higher yields (86%) of DHPM in a shorter time (55 min) than upon conventional heating, which suggested synergism between microwaves and the enzyme. Barsode et al. investigated the impact of microwave radiation on the synthesis of isoamyl butyrate ester catalyzed by lipase [90]. Microwave radiation remarkably decreased the activation energy of the reaction, showing that microwave irradiation can accelerate the formation of the transition state of enzyme substrates to improve the reaction rate.

Notably, ionic liquids (ILs), as a greener alternative to organic solvents [91,92], are ideal solvents for enzyme-catalyzed reactions under microwave radiation because of their excellent microwave-absorbing ability, high boiling point, and low vapor pressure [93]. Currently, a lipase-mediated, microwave-assisted anthocyanin lipophilization with ILs, which can improve the biomedical properties of anthocyanins, including the anti-inflammatory and antioxidant properties, has been reported for the first time [94,95]. Microwaves significantly reduced the reaction time and achieved maximum product generation. On the other hand, ILs comprised of imidazolium-based cations and triflate anions showed better performance in terms of improving the substrate solubility and stability and activity of the biocatalyst. Moreover, the combination of ILs and microwaves exhibited synergistic effects for enzymatic synthesis of biodiesel production, improving enzyme activity and reusability of enzymes and ILs [13]. Overall, these promising results demonstrated that microwave radiation and ILs involved in enzyme reactions are a prospective strategy that can be applied to biomedicine and industrial production.

Food industry: Applications to food industrial fields are expected, as microwave radiation has excellent properties, including efficient heating and energy consumption [96]. Specifically, microwaves can break noncovalent molecules of food to change the physicochemical properties, contribute to the pretreatment of food, and have a synergistic effect with enzymes in food processing. It was reported that transglutaminase (TGase) could be used to enhance the cross-linking of myofibrils of surimi, which can improve the gel strength of surimi products [97]. Based on this, Cao et al. explored the effects of TGase on the gel properties of surimi under microwave radiation [98,99]. Microwave-assisted Tgase remarkably improved gel strength by catalyzing covalent cross-linking of proteins to form a compact and uniform net structure. Furthermore, microwave-accelerated cellulase can catalyze chitosan hydrolysis into low molecular weight chitosan, which has been widely applied in the food industry, agriculture, and biomedicine [100,101]. Therefore, many studies have confirmed that microwave radiation can be utilized extensively for either enzyme-catalyzed chemical reactions, the food industry, or other application fields.

## 4. Ultrasound Strategy

### 4.1. Mechanism

Ultrasound refers to sound waves with frequencies ranging from 20 kHz to 5 MHz and has received remarkable attention due to its great directionality, strong penetrating ability, and concentrated sound energy. Ultrasound can generate cavitation phenomena and bubble collapse processes that eliminate mass transfer resistance, improve the transport process and intensively increase the pressure and temperature as well as the intense shear forces inside the bubble [102,103]. Based on this, ultrasound has been widely applied to improve the rates of many chemical, physical, and biological processes, including enzyme-catalyzed bioprocesses. Over the past decades, many reviews have discussed the effect of ultrasound irradiation on enzyme properties [104,105,106]. Firstly, the mechanism of ultrasound-activated enzyme activity is that the consecutive wave and cavitation bubbles upon ultrasound can make favorable conformational changes by altering the loop and domain regions of the enzyme to improve enzymatic properties [104]. Similar to the microwave strategy, ultrasound energy can change the proportion of the enzymatic secondary structure, including α-helices, β-sheets, β-turns, and random coils, depending on the characteristics of the enzyme and ultrasound parameters [107,108,109]. It has been reported that these conformational changes could reduce the activation energy because of the available release of the substrate from the active site of the enzyme, thereby improving catalytic efficiency and enzyme activity [110]. Jadhav et al. has confirmed that the conformational changes influenced enzyme kinetics by improving the reaction rate by 1.45 fold upon ultrasound [111]. Furthermore, after ultrasound treatment, the enzyme kinetics exhibited an increment in V_max_ and reduction in K_m_, which enhanced the affinity between enzyme and substrate. Besides, the hydrodynamic shear force due to the quick breakdown of microbubbles generated by the cavitation effect can depose large materials into small particles, which extends the surface area of substrates for the enzymatic attack, improves the transport phenomenon, and diminishes mass transfer resistance, thereby enhancing the catalytic performance of enzymes. Moreover, ultrasonic shockwaves produced by the sudden collapse of microbubbles also improve the stability of enzymes and inhibit harmful protein aggregation [112]. It was worth noting that cavitation plays a different role in enzyme-mediated heterogeneous and homogeneous systems. In heterogeneous systems cavitation acts mainly through physical effects and mainly chemical effects in homogeneous systems [113]. These studies demonstrated that ultrasound irradiation could be applied as an effective protocol for enzyme activation.

**Figure 8:**
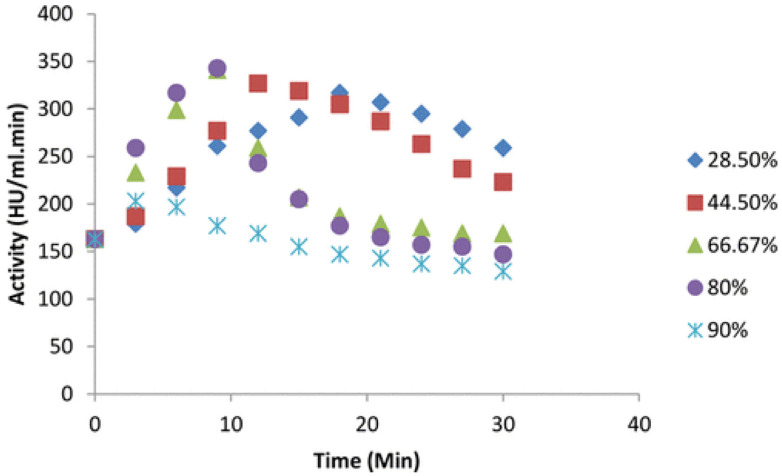
The change of enzyme activity with different duty cycles of ultrasound irradiation in 30 min (intensity: 12.22 W/cm^2^, enzyme concentration: 3.0 g/mL). Reprinted with permission from Jadhav et al., American Chemical Society [111].

Effect of Parameters: The parameters of ultrasound irradiation, including the frequency, intensity/power, amplitude, and duty cycle, play an important role in enzymatic properties and activity. First, ultrasound frequency can influence cavitation bubble collapse, which determines active or inactive conformational changes in enzymes. Many studies have confirmed that a higher frequency could decrease enzyme activity or even denature the enzyme because of the dramatically rising temperature caused by bubble collapse [112,113,114]; when cellulase is exposed to an optimum ultrasound frequency, favorable conformational changes [115]. Furthermore, ultrasound intensity may induce a cavitation effect and mechanical oscillation, which affect the rearrangement of hydrogen bonds or van der Waals forces [111,116]. However, high intensity can generate free hydroxyl and hydrogen radicals, which destroy the enzyme structure and denature the Lipozyme 435. In addition, the duty cycle of irradiation is also one of the important parameters for controlling enzyme activity. The duty cycle often influences the exposure time of enzymes to ultrasound and energy consumption. It was worth noting that continuous ultrasound might cause damage to the structure of lipase CALB due to the excessive heat induced by the cavitation effect (Figure 8) [111]. Overall, enzyme activity and conformation stability are sensitive to ultrasound parameters; thus, optimizing the conditions of ultrasound parameters is necessary to obtain highly active enzymes.

Therefore, as an effector of such local variation, ultrasounds show significant benefits, such as enhanced selectivity and lower energy consumption, and have the potential to influence the activity of enzymatic reactions. However, the stability of enzymes is highly required when using ultrasounds for enzyme activation since the high energy input might occasionally inactivate the enzyme.

### 4.2. Applications

Ultrasound-assisted enzyme reactions with optimal parameters have been proposed in food processing and preservation, as well as for industrial applications. For example, Soares et al. explored lipase-catalyzed goat cream hydrolysis under ultrasound to obtain short-chain fatty acids that could be used for flavoring in the food industry [12]. It was observed that ultrasound-pretreated lipase promoted cream hydrolysis at lower temperatures than nontreated lipase, which could reduce undesirable thermal effects and make it available for industrial processing. Furthermore, the proper ultrasound has improved the activity of cellulase to hydrolyze brown rice with increased kinetics parameters and favorable conformational change in order to obtain high qualities and productivities [117]. This result also confirmed that sonicated cellulase exhibited prospective application as an efficient technique in whole-grain processing. Amyloglucosidase (AMG) can be applied for starch saccharification to produce glucose for different industries, including the production of perfumes, medicines, and alcoholic beverages. It has been confirmed that ultrasound was able to improve the activity and stability of AMG at nonoptimal pH and temperature, expanding the scope of industrial applications of AMG [118]. Another example was that ultrasound promoted xylanase to catalyze the hydrolysis of xylan to xylooligosaccharides, which can stimulate the growth of beneficial bacteria, maintain gastrointestinal health, and improve the bioavailability of calcium [119]. The results showed that the combination of enzymes and ultrasound had a synergistic effect on xylan hydrolysis, increasing the reducing sugar content by approximately 50%. The existence of cetirizine in wastewater can pose a threat to aquatic c ecosystems, including endocrine disruption and other hazardous side effects. Notably, ultrasound-assisted enzymes can also be applied to biomedicine yield. In addition to food processing, ultrasound irradiation has been explored in the lipase-catalyzed synthesis of propyl caprate, which was widely used as a fragrant compound and an intermediate for drug molecules preparation. With the optimum parameters of ultrasound, the synthesis efficiency catalyzed by lipase has been enhanced in a shorter time. Moreover, the stability and reusability of the enzyme also increased upon ultrasound compared to conventional synthesis. Thus, ultrasound application showed great potential to improve the performance of biocatalyst in this esterification reaction [120]. Many studies have demonstrated that ultrasound has great potential in enzymatic industrial applications because it can improve enzyme properties and catalytic activity under optimal conditions.

## 5. Alternating Magnetic Field Strategy

### 5.1. Mechanism

Over the past decades, the application of alternating magnetic fields has been a new trend in enzyme activation due to their deep tissue penetration and precise on-off control. There are two strategies to activate enzymatic bioprocesses under AMF [121]. One is to control the activity of the enzyme by influencing the enzymatic structure containing iron cofactors such as heme [122,123]. Another strategy is to immobilize the enzyme on magnetic nanomaterials and then utilize the nanoscale effect of the particles under the AMF to control the activity of the enzyme [124]. However, most studies have concentrated on the latter strategy, and only a few studies have investigated the effect of direct AMF-enzyme activation because of the limited type of enzyme-containing iron cofactors such as horseradish peroxidase (HRP).

Enzyme with iron cofactors: Studies concerning the effects of magnetic fields on enzyme activities have been reported [125,126,127]. External magnetic fields can cause conformational changes in proteins by interacting with specific ions, including magnesium, manganese, calcium, or iron, as specific cofactors [128]. Many reactions concerning charge transfer catalyzed by oxidoreductase can be promoted by a magnetic field, which can potentially control enzyme catalytic properties. Many studies have observed that HRP activity, with heme as a cofactor, is significantly dependent on the characteristics of the magnetic field. The catalytic center of HRP consists of Fe^3+^ in the hem group and two calcium ions, which are important for enzyme function and conformation. When exposed to a magnetic field, the reduced form of the heme may exhibit paramagnetic or diamagnetic properties [129,130]. Therefore, it is proposed that a magnetic field can promote charge transfer in the active center, improving the rate of the enzymatic reaction.

Combination enzyme with magnetic nanoparticles: Recently, magnetic nanoparticles, due to their high specific surface area, good biocompatibility and dispersion, simple separation, and easy modification have been widely used for enzyme immobilization to control the activity of enzymes upon AMF [131,132,133,134,135,136,137]. Magnetic nanoparticles occur molecular vibrations due to the changing magnetic field, which depends on the frequency of the magnetic field. Under a lower magnetic frequency, magnetic nanoparticles oscillate and perform similar to microscopic stirrers to facilitate mass transfer processes, accelerating the reaction rate. Xia et al. found that when exposed to an AMF frequency of 600 Hz, the reaction rate of Fe_3_O_4_-NH_2_-PEI (1200)-laccase increased approximately 1.16 times greater than conventional mechanical stirring [134]. The results also demonstrate that increasing the frequency ranging from 50 to 600 Hz induces a rapid direction change of the enzyme-nanoparticle complex, which enhances the mobility of Fe_3_O_4_-NH_2_-PEI (1200)-laccase and thus increases the reaction rate. Liu et al. reported that magnetic cross-linked lipase aggregates (MCLEAs), acting as microscopic stirrers under AMF, can effectively improve the resolution of (R,S)-2-octanol [137].

**Figure 9:**
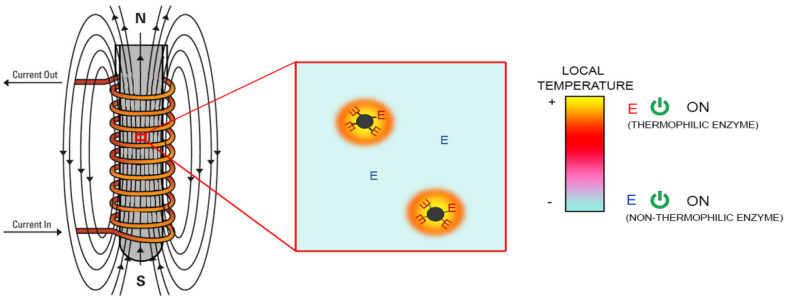
The procedure of thermophilic enzymes-Fe_3_O_4_ nanocomposites activated by local magnetothermal effect upon AMF due to the molecular vibration of MNPs. The non-thermophlic enzyme without immobilized on Fe_3_O_4_ nanoparticles can work together with the thermophilic enzymes-Fe_3_O_4_ nanocomposites in the same reaction due to the local magnetothermal effect. Adapted with permission from Armenia et al., Elsevier [135].

For the higher magnetic frequency, the magnetothermal effect of magnetic particles occurs due to Neel or Brown relaxation loss. The Brown relaxation loss comes from the friction between rotating magnetic nanoparticles and the liquid under the AMF, and the Neel relaxation loss comes from the magnetic moment changing in the nanoparticles with the direction changing the external field [133]. Notably, the magnetic-induced heat effect, unlike the photothermal effect caused by NIR, only elevates the local temperature on the nanoscale rather than increasing the environmental temperature in the solution. For example, Xiong et al. covalently bound β-galactosidase (β-Gal) to a ferrimagnetic vortex-domain nanoring (FVIO), and they observed that enzyme activity could be stimulated by local heating on the surface of FVIO under an AMF of 345 kHz in almost real-time [124]. Furthermore, two thermophilic enzymes, α-amylase (AMY) and L-aspartate oxidase (LASPO), immobilized on iron oxide NPs through four different conjunction methods, were efficiently activated by an AMF of 410–829 kHz (Figure 9) [135]. It was also observed that the localization of the enzyme molecules on the NP surface is critical to maximizing this activation effect. This may be the result of different heat transfer mechanisms and the different rigidities of the enzyme in the region attached to the carrier. The kinetic parameters of enzymes also can be affected by the magnetothermal effect. For example, Knecht et al. incorporated Fe_3_O_4_ nanoparticles and a thermophilic dehalogenase into a bisacrylamide cross-linked polyacrylamide hydrogel network and explored the kinetic change in the presence of an AMF [132]. It was found that the Km decreased due to the improved affinity between enzymes and substrates when heated with an AMF, and the Kcat increased because of the heat-induced higher product turnover. Additionally, the selective heating of AMF provides a potential application allowing other nonthermophilic enzymes to work together with thermophilic enzymes.

These studies confirmed that enzyme activity could be spatiotemporally and precisely activated due to the thermal effect on the surface of magnetic particles under AMF. Although the frequency of AMF can affect the mechanism of activation, there is still no definite division of the low- or high-frequency range. Sometimes, the enzyme activity could not be greatly increased (approximately two-fold) because of the poor magnetothermal conversion efficiency. In addition to improving enzyme activity, it has been reported that magnetic fields can also be used in biological macromolecule assembly. For instance, HRP molecule assembly can be activated by AMF due to the high magnetization, which suggests the feasibility of molecular assembly by magnetic interactions [138]. Under stimulation by AMF, the HRP molecules oscillate with the external field and then induce antiparallel magnetic moments, which can generate anisotropic and attractive interactions. This provides a promising potential for the application of AMF-controlled biomolecule assembly techniques.

### 5.2. Applications

Industrial applications: Over the past few years, AMF-mediated enzyme activation technology has been widely applied in industrial applications such as the pharmaceutical industry, degradation of contaminants, and the production of industrial raw material [134,135,136,139]. Phenolic compounds are troublesome pollutants produced by a variety of industries. Xia et al. immobilized laccase on polyethyleneimine (PEI)-modified amine-functionalized Fe_3_O_4_ nanoparticles, and the oxidation rate of catechol was enhanced under AMF [134]. Moreover, another work reported by the same group engineered a strategy by constructing a newly fixed bed reactor and achieved the continuous degradation of phenolic compounds at a high gradient magnetic field [139]. They further investigated the different impacts between continuous and batch treatment, finding that the rate of continuous treatment on the bed for 18 h was 2.38 times higher than batch treatment for six cycles. It is worth noting that the degradation rate was maintained at over 70% within 48 h when treated with Fe_3_O_4_-NH_2_-PEI laccase in the fixed bed reactor, which showed great potential for the continuous degradation of phenol compounds in industrial wastewater. In addition to its application in contamination control, AMF-mediated enzyme activation is also used to produce industrial raw materials. Cui et al. designed a three-phase fluidized bed reactor with magnetically immobilized cellulase that can produce chitooligosaccharides from chitosan [140]. They found an apparent increase in chitooligosaccharide production under AMF, which is very attractive in the food and pharmaceutical industries [141].

Moreover, the resolution of optical enantiomers of many organic compounds plays an important role in producing medicine, agriculture, fragrances, and flavors. Enzymes with high stereo-regioselectivity are widely used in catalyzing preferable and worthwhile enantiomers, which is called enzymatic kinetic resolution [136,142,143]. Liu et al. constructed magnetic cross-linked lipase aggregates (MCLEAs), which revealed an efficient enhancement in separating (R,S)-2-octanol under AMF [137]. AMF-mediated enzyme activation shows promising potential in enhancing the reaction rate as well as enzyme stability in enzymatic kinetic resolution, and it is worth further exploration in the pharmaceutical industry. 

**Figure 10:**
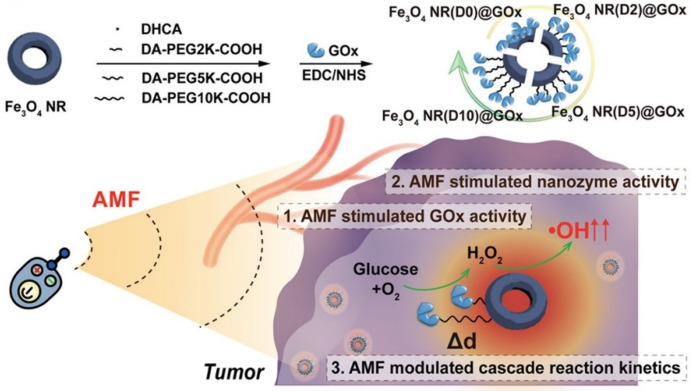
Fe_3_O_4_ NR@GOx nanocatalysts used for tumor therapy under AMF. The top shows the assembly process of Fe_3_O_4_ NR@GOx nanocatalysts, and the bottom shows their effect on tumor therapy. Reprinted with permission from Zhang et al., American Chemical Society [144].

Biomedicine applications: AMF-activated enzyme-magnetic nanomaterial composites can also be applied to achieve multifunctional tumor therapy strategies. Fan et al. constructed a magnetic field-based platform by immobilizing glucose oxidase (GOx) on a ferrimagnetic vortex iron oxide nanoring (Fe_3_O_4_ NR) for tumor therapy (Figure 10) [144]. Notably, Fe_3_O_4_ NRs can not only be used as immobilized supports of enzymes but also exhibits peroxidase nanozyme activity that can transform hydrogen peroxide generated by GOx into •OH to implement cascade reactions. These reactive oxygen species (ROS) molecules induce cell death and tissue destruction and are the main cytotoxic species that kill tumor cells. The results showed that AMF could precisely activate and enhance the activity of Fe_3_O_4_ NR@GOx to produce significant ROS due to AMF-induced heat conversion in a distance-dependent manner. Thus, Fe_3_O_4_ NR@GOx has a remarkable improvement in the tumor suppression effect when exposed to AMF. The construction of magneto-responsive enzyme−nanozyme cascade catalysts is a promising platform that could contribute to multifunctional cancer therapy and even metabolic processes in living organisms.

## 6. Conclusions

This review summarizes the recent progress made in real-time activation strategies, highlighting the significant role of controlling enzyme activity. NIR strategy can activate enzyme activity by combining biocatalysts with plasmonic nanomaterials, which generate a photothermal effect or electron transfer stimulated by light to improve activity. Microwave and ultrasound irradiation generally alter enzymatic structure into favorable and flexible conformation by influencing noncovalent interaction to increase the stability, selectivity, or kinetics of enzymes. However, further studies with respect to the mechanism of these two activation strategies are necessary to carry out since a number of different types of enzymes are not responsive to microwave and ultrasound, and there are no reasonable studies and discussions. AMF can activate not only some special oxidoreductases such as HRP but also enhance the potency of enzymes immobilized on magnetic nanomaterials due to the local heating on the surface. Moreover, the dimensions, morphology of nanoparticles, intensity, frequency of real-time activation strategies, and reaction solvents have remarkable impacts on controlling enzyme activity. Hence, parameter optimization is obviously significant when applying these activation strategies to improve enzymatic properties. It is known that remote-controlled enzyme technologies have been widely used in the chemical synthesis, biomedical, and food industries. Furthermore, the combination of different real-time activation strategies may potentially become a novel prospective protocol to exert synergistic effects for maximizing enzyme activity [145].

## Figures and Tables

**Table 1:** NIR-responsive nanoparticles for immobilized enzyme and their applications.

Material	Enzyme	Application	Ref.
Gold nanorod	Dextran hydrolase, glucose oxidase, horseradish peroxidase	Microreactor	[22]
Polydopamine-coated gold nanorods	Papain	Deep-tumor therapy	[10]
Gold nanorods	Acylpeptide hydrolase ST0779	Alzheimer’s disease therapy	[41]
Gold nanoparticles	Bovine pancreatic ribonuclease	Colon cancer therapy	[34]
Ultrasmall platinum nanoparticle	Glucoamylase (GA), ProteinaseK, Deoxyribonuclease I	Off-on control of enzyme activity.	[42]
Ti_3_C_2_T_X_ nanosheets	Lipase	Improve the hydrolysis activity	[43]
Gold nanorod	Acylpeptide hydrolase, Pig pancreatic lipase	Aldol reaction	[20]
Gold nanoparticle	Alkaline phosphatase	Prostate cancer therapy	[35]
Graphene oxide	Horseradish peroxidase	Colorimetric immunoassay	[44]
CdS	Nitrogenase MoFe	Dinitrogen reduction	[18]

## Data Availability

No new data were created or analyzed in this study. Data sharing is not applicable to this article.

## References

[1] Madhavan A., Sindhu R., Binod P. (2017). Strategies for design of improved biocatalysts for industrial applications. Bioresour. Technol..

[2] Li S., Yang X., Yang S. (2012). Technology prospecting on enzymes: Application, marketing and engineering. Comput. Struct. Biotec..

[3] Wu S., Snajdrova R., Moore J.C., Baldenius K., Bornscheuer U.T. (2021). Biocatalysis: Enzymatic Synthesis for Industrial Applications. Angew. Chem. Int. Ed..

[4] Arbige M.V., Shetty J.K., Chotani G.K. (2019). Industrial Enzymology: The Next Chapter. Biotechnol. Trends. Biotechnol..

[5] Zhang Z., Zheng B., Wang Y. (2008). The conserved N-terminal helix of acylpeptide hydrolase from archaeon Aeropyrum pernix K1 is important for its hyperthermophilic activity. BBA Proteins. Proteom..

[6] Rueda N., Dos Santos J.C.S., Ortiz C. (2016). Chemical modification in the design of immobilized enzyme biocatalysts: Drawbacks and opportunities. Chem. Rec..

[7] Nguyen H.H., Lee S.H., Lee U.J. (2019). Immobilized enzymes in biosensor applications. Materials.

[8] Zhou Z., Hartmann M. (2013). Progress in enzyme immobilization in ordered mesoporous materials and related applications. Chem. Soc. Rev..

[9] Borzouee F., Varshosaz J., Cohan R.A. (2021). A Comparative Analysis of Different Enzyme Immobilization Nanomaterials: Progress, Constraints, and Recent Trends. Curr. Med. Chem..

[10] Wu D., Chen X., Zhou J. (2020). A synergistic optical strategy for enhanced deep-tumor penetration and therapy in the second near-infrared window. Mater. Horiz..

[11] Wang F., Owusu-Fordjour M., Xu L. (2020). Immobilization of laccase on magnetic chelator nanoparticles for apple juice clarification in magnetically stabilized fluidized bed. Front. Bioeng. Biotech..

[12] Soares A.D., Leite B.R.D., Tribst A.A.L., Augusto P.E.D., Ramos A.M. (2020). Effect of ultrasound on goat cream hydrolysis by lipase: Evaluation on enzyme, substrate and assisted reaction. LWT Food Sci. Technol..

[13] Yu D., Wang C., Yin Y. (2011). A synergistic effect of microwave irradiation and ionic liquids on enzyme-catalyzed biodiesel production. Green. Chem..

[14] De Barros H.R., Garcia I., Kuttner C. (2020). Mechanistic insights into the light-driven catalysis of an immobilized lipase on plasmonic nanomaterials. ACS Catal..

[15] Mosquera J., Zhao Y., Jang H.J. (2020). Plasmonic nanoparticles with supramolecular recognition. Adv. Funct. Mater..

[16] Liz-Marzán L.M., Murphy C.J., Wang J. (2014). Nanoplasmonics. Chem. Soc. Rev..

[17] Linic S., Aslam U., Boerigter C. (2015). Photochemical transformations on plasmonic metal nanoparticles. Nat. Mater..

[18] Brown K.A., Harris D.F., Wilker M.B. (2016). Light-driven dinitrogen reduction catalyzed by a CdS: Nitrogenase MoFe protein biohybrid. Science.

[19] Guo S., Li H., Liu J. (2015). Visible-light-induced effects of Au nanoparticle on laccase catalytic activity. ACS Appl. Mater. Interfaces.

[20] Li W., Liu D., Geng X. (2019). Real-time regulation of catalysis by remote-controlled enzyme-conjugated gold nanorod composites for aldol reaction-based applications. Catal. Sci. Technol..

[21] Tadepalli S., Yim J., Madireddi K. (2017). Gold nanorod-mediated photothermal enhancement of the biocatalytic activity of a polymer-encapsulated enzyme. Chem. Mater..

[22] Su J., Chen H., Xu Z. (2020). Near-Infrared-Induced Contractile Proteinosome Microreactor with a Fast Control on Enzymatic Reactions. ACS Appl. Mater. Interfaces.

[23] Tadepalli S., Yim J., Cao S. (2018). Metal–organic framework encapsulation for the preservation and photothermal enhancement of enzyme activity. Small.

[24] Thompson S.A., Paterson S., Azab M.M.M. (2017). Light-Triggered Inactivation of Enzymes with Photothermal Nanoheaters. Small.

[25] Blankschien M.D., Pretzer L.A., Huschka R. (2013). Light-triggered biocatalysis using thermophilic enzyme–gold nanoparticle complexes. Acs Nano.

[26] Kang P., Chen Z., Nielsen S.O. (2017). Molecular hyperthermia: Spatiotemporal protein unfolding and inactivation by nanosecond plasmonic heating. Small.

[27] Bretschneider J.C., Reismann M., von Plessen G., Simon U. (2009). Photothermal Control of the Activity of HRP-Functionalized Gold Nanoparticles. Small.

[28] Wang C., Zhang Q., Wang X. (2017). Dynamic Modulation of Enzyme Activity by Near-Infrared Light. Angew. Chem. Int. Ed..

[29] Hedison T.M., Heyes D.J., Scrutton N.S. (2022). Making molecules with photodecarboxylases: A great start or a false dawn. Curr. Res. Chem. Biol..

[30] Weber S. (2005). Light-driven enzymatic catalysis of DNA repair: A review of recent biophysical studies on photolyase. Biochim. Biophys. Acta Bioenerg..

[31] Sorigué D., Légeret B., Cuiné S., Blangy S., Moulin S., Billon E., Richaud P., Brugière S., Couté Y., Nurizzo D. (2017). An algal photoenzyme converts fatty acids to hydrocarbons. Science.

[32] Heyes D.J., Zhang S., Taylor A., Johannissen L.O., Hardman S.J.O., Hay S., Scrutton N.S. (2021). Photocatalysis as the ‘master switch’ of photomorphogenesis in early plant development. Nativ. Plants.

[33] Emmanuel M.A., Greenberg N.R., Oblinsky D.G., Hyster T.K. (2016). Accessing non-natural reactivity by irradiating nicotinamide-dependent enzymes with light. Nature.

[34] Khiavi M.A., Safary A., Aghanejad A. (2019). Enzyme-conjugated gold nanoparticles for combined enzyme and photothermal therapy of colon cancer cells. Colloid. Surface. A.

[35] Yang S., Yao D., Wang Y., Yang W., Zhang B., Wang D. (2018). Enzyme-triggered self-assembly of gold nanoparticles for enhanced retention effects and photothermal therapy of prostate cancer. Chemical Communications. Chem. Commun..

[36] Sun M., Xu H. (2012). A novel application of plasmonics: Plasmon-driven surface-catalyzed reactions. Small.

[37] Breger J.C., Oh E., Susumu K. (2019). Nanoparticle size influences localized enzymatic enhancement—A case study with Phosphotriesterase. Bioconjugate Chem..

[38] Burda C., Chen X., Narayanan R. (2005). Chemistry and properties of nanocrystals of different shapes. Chem. Rev..

[39] Xia Y., Halas N.J. (2005). Shape-controlled synthesis and surface plasmonic properties of metallic nanostructures. MRS Bull..

[40] Ye T., Dai Z., Mei F. (2016). Synthesis and optical properties of gold nanorods with controllable morphology. J. Phys. Condens. Mat..

[41] Liu D., Li W., Jiang X., Bai S., Liu J., Liu X., Shi Y., Kuai Z., Kong W., Gao R. (2019). Using near-infrared enhanced thermozyme and scFv dual-conjugated Au nanorods for detection and targeted photothermal treatment of Alzheimer’s disease. Theranostics.

[42] Zhang S., Wang C., Chang H. (2019). Off-on switching of enzyme activity by near-infrared light-induced photothermal phase transition of nanohybrids. Sci. Adv..

[43] Ding C., Liang J., Zhou Z. (2019). Photothermal enhanced enzymatic activity of lipase covalently immobilized on functionalized Ti_3_C_2_TX nanosheets. Chem. Eng. J..

[44] Cao G., Sun D., Gu T. (2019). Photoswitching enzymatic activity of horseradish peroxidase by graphene oxide for colorimetric immunoassay. Biosens. Bioelectron..

[45] Kamada K. (2019). Photo-manipulation of activity of enzymes bound to inorganic nanomaterials. J. Solid State Chem..

[46] Sheng S., Liu F., Lin L. (2020). Nanozyme-mediated cascade reaction based on metal-organic framework for synergetic chemo-photodynamic tumor therapy. J. Control. Release.

[47] Bai J., Liu Y., Jiang X. (2014). Multifunctional PEG-GO/CuS nanocomposites for near-infrared chemo-photothermal therapy. Biomaterials.

[48] Zhang L., Su H., Cai J. (2016). A multifunctional platform for tumor angiogenesis-targeted chemo-thermal therapy using polydopamine-coated gold nanorods. ACS Nano.

[49] Wang Y., Wang K., Zhao J. (2013). Multifunctional mesoporous silica-coated graphene nanosheet used for chemo-photothermal synergistic targeted therapy of glioma. J. Am. Chem. Soc..

[50] Kim N., Lee H.J. (2020). Target Enzymes Considered for the Treatment of Alzheimer’s Disease and Parkinson’s Disease. Biomed. Res. Int..

[51] Fuentes-Baile M., García-Morales P., Pérez-Valenciano E. (2020). Cell Death Mechanisms Induced by CLytA-DAAO Chimeric Enzyme in Human Tumor Cell Lines. Int. J. Mol. Sci..

[52] Jiang Y., Li J., Zhen X. (2018). Dual-peak absorbing semiconducting copolymer nanoparticles for first and second near-infrared window photothermal therapy: A comparative study. Adv. Mater..

[53] Zhou J., Jiang Y., Hou S. (2018). Compact plasmonic blackbody for cancer theranosis in the near-infrared II window. Acs Nano.

[54] Chen X., Chen Y., Xin H. (2020). Near-infrared optogenetic engineering of photothermal nanoCRISPR for programmable genome editing. Proc. Natl. Acad. Sci. USA.

[55] Zhao J., Fei J., Du C. (2013). Assembly of catalase-based bioconjugates for enhanced anticancer efficiency of photodynamic therapy in vitro. Chem. Commun..

[56] Cho Y., Kim H., Choi Y. (2013). A graphene oxide–photosensitizer complex as an enzyme-activatable theranostic agent. Chem. Commun..

[57] Du B., Tung C.H. (2020). Enzyme-assisted photodynamic therapy based on nanomaterials. ACS Biomater. Sci. Eng..

[58] Chen Q., Chen J., Yang Z. (2018). NIR-II light activated photodynamic therapy with protein-capped gold nanoclusters. Nano Res..

[59] Hamley I.W. (2012). The amyloid beta peptide: A chemist’s perspective. Role in Alzheimer’s and fibrillization. Chem. Rev..

[60] Viola K.L., Klein W.L. (2015). Amyloid β oligomers in Alzheimer’s disease pathogenesis, treatment, and diagnosis. Acta Neuropathol..

[61] Li M., Howson S.E., Dong K. (2014). Chiral metallohelical complexes enantioselectively target amyloid β for treating Alzheimer’s disease. J. Am. Chem. Soc..

[62] He G., Luo W., Li P., Remmers C., Netzer W.J., Hendrick J. (2010). Gamma-secretase activating protein is a therapeutic target for Alzheimer’s disease. Nature.

[63] Pan S., Ren J., Ma E., Wang K., Yang S., Wang H. (2021). Dual-Propelled Sporopollenin-Exine-Capsule Micromotors for Near-Infrared Light Triggered Degradation of Organic Pollutants. Chem. Nano Mat..

[64] Banik S., Bandyopadhyay S., Ganguly S. (2003). Bioeffects of microwave—A brief review. Bioresour. Technol..

[65] Horikoshi S., Serpone N. (2019). Microwave flow chemistry as a methodology in organic syntheses, enzymatic reactions, and nanoparticle syntheses. Chem. Rec..

[66] Wang Z., Zhang Y., Zheng L., Cui X., Huang H., Geng X., Xie X. (2018). Regioselective acylation of resveratrol catalyzed by lipase under microwave. Green Chem. Lett. Rev..

[67] Khan N.R., Rathod V.K. (2020). Microwave mediated lipase-catalyzed synthesis of n-butyl palmitate and thermodynamic studies. Biocatal. Agric. Biotechnol..

[68] Mazinani S.A., DeLong B., Yan H. (2015). Microwave radiation accelerates trypsin-catalyzed peptide hydrolysis at constant bulk temperature. Tetrahedron Lett..

[69] Zhang X., Cao T., Tian X. (2012). Effect of microwave irradiation on the structure of glucoamylase. Process Biochem..

[70] Yu D., Wang Y., Wang C. (2012). Combination use of microwave irradiation and ionic liquid in enzymatic isomerization of xylose to xylulose. J. Mol. Catal. B Enzym..

[71] Liu N., Wang L., Wang Z. (2015). Microwave-assisted resolution of α-lipoic acid catalyzed by an ionic liquid co-lyophilized lipase. Molecules.

[72] Shinde S.D., Yadav G.D. (2015). Insight into microwave-assisted lipase catalyzed synthesis of geranyl cinnamate: Optimization and kinetic modeling. Appl. Biochem. Biotech..

[73] Rokhati N., Pramudono B., Istirokhatun T. (2018). Microwave Irradiation-Assisted Chitosan Hydrolysis Using Cellulase Enzyme. Bull. Chem. React. Eng..

[74] Siguemoto É.S., Pereira L.J., Gut J.A.W. (2018). Inactivation kinetics of pectin methylesterase, polyphenol oxidase, and peroxidase in cloudy apple juice under microwave and conventional heating to evaluate non-thermal microwave effects. Food Bioprocess Technol..

[75] Jiao X., Fan D. (2022). Non-thermal microwave effects: Conceptual and methodological problems. Food Chem..

[76] Gutmann B., Schwan A.M., Reichart B. (2011). Activation and deactivation of a chemical transformation by an electromagnetic field: Evidence for specific microwave effects in the formation of Grignard reagents. Angew. Chem. Int. Ed..

[77] Dudley G.B., Stiegman A.E., Rosana M.R. (2013). Correspondence on microwave effects in organic synthesis. Angew. Chem. Int. Ed..

[78] Kappe C.O. (2013). Reply to the correspondence on microwave effects in organic synthesis. Angew. Chem. Int. Ed..

[79] Nagashima I., Sugiyama J., Sakuta T. (2014). Efficiency of 2.45 and 5.80 GHz microwave irradiation for a hydrolysis reaction by thermostable β-Glucosidase HT1. Biosci. Biotech. Bioch..

[80] Young D.D., Nichols J., Kelly R.M. (2008). Microwave activation of enzymatic catalysis. J. Am. Chem. Soc..

[81] Kubo M.T.K., Siguemoto E.S., Funcia E.S. (2020). Non-thermal effects of microwave and ohmic processing on microbial and enzyme inactivation: A critical review. Curr. Opin. Food Sci..

[82] Yang J., Chen X., Yu D. (2016). Microwave-assisted synthesis of butyl galactopyranoside catalyzed by β-galactosidase from *Thermotoga naphthophila* RKU-10. Process Biochem..

[83] Kappe C.O. (2004). Controlled microwave heating in modern organic synthesis. Angew. Chem. Int. Ed..

[84] Meriles S.P., Steffolani M.E., Penci M.C., Curet S., Boillereaux L., Ribotta P.D. (2021). Effects of low-temperature microwave treatment of wheat germ. J. Sci. Food Agric..

[85] Jaiswal K.S., Rathod V.K. (2021). Process Intensification of Enzymatic Synthesis of Flavor Esters: A Review. Chem. Rec..

[86] Dill L.P., Kochepka D.M., Krieger N. (2019). Synthesis of fatty acid ethyl esters with conventional and microwave heating systems using the free lipase B from Candida antarctica. Biocatal. Biotransfor..

[87] Xie Z.B., Fu L.H., Meng J. (2020). Efficient biocatalytic strategy for one-pot Biginelli reaction via enhanced specific effects of microwave in a circulating reactor. Bioorg. Chem..

[88] Yu D., Ma D., Wang Z. (2012). Microwave-assisted enzymatic resolution of (R,S)-2-octanol in ionic liquid. Process Biochem..

[89] Klibanov A.M. (2001). Improving enzymes by using them in organic solvents. Nature.

[90] Bansode S.R., Rathod V.K. (2018). Enzymatic sythesis of Isoamyl butyrate under microwave irradiation. Chem. Eng. Process..

[91] Capela E.V., Valente A.I., Nunes J.C.F. (2020). Insights on the laccase extraction and activity in ionic-liquid-based aqueous biphasic systems. Sep. Purif. Technol..

[92] Zhang Y., Wang N., Xie Z.B. (2014). Ionic liquid as a recyclable and efficient medium for lipase-catalyzed asymmetric cross aldol reaction. J. Mol. Catal. B Enzym..

[93] Zhao H., Baker G.A., Song Z. (2009). Effect of ionic liquid properties on lipase stabilization under microwave irradiation. J. Mol. Catal. B Enzym..

[94] Guimarães M., Mateus N., de Freitas V. (2020). Microwave-Assisted Synthesis and Ionic Liquids: Green and Sustainable Alternatives toward Enzymatic Lipophilization of Anthocyanin Monoglucosides. J. Agric. Food Chem..

[95] Novilla A., Djamhuri D.S., Nurhayati B. (2017). Anti-inflammatory properties of oolong tea (*Camellia sinensis*) ethanol extract and epigallocatechin gallate in LPS-induced RAW 264.7 cells. Asian. Pac. J. Trop. Biomed..

[96] Guo Q., Sun D.W., Cheng J.H. (2017). Microwave processing techniques and their recent applications in the food industry. Trends Food Sci. Technol..

[97] Grossmann L., Wefers D., Bunzel M. (2017). Accessibility of transglutaminase to induce protein crosslinking in gelled food matrices-Influence of network structure. LWT Food Sci. Technol..

[98] Cao H.W., Fan D., Jiao X. (2018). Intervention of transglutaminase in surimi gel under microwave irradiation. Food Chem..

[99] Cao H., Jiao X., Fan D. (2019). Catalytic effect of transglutaminase mediated by myofibrillar protein crosslinking under microwave irradiation. Food Chem..

[100] Prashanth K.V.H., Tharanathan R.N. (2007). Chitin/chitosan: Modifications and their unlimited application potential—An overview. Trends Food Sci. Technol..

[101] Huang K.S., Wu W.J., Chen J.B. (2008). Application of low-molecular-weight chitosan in durable press finishing. Carbohydr. Polym..

[102] Gogate P.R., Kabadi A.M. (2009). A review of applications of cavitation in biochemical engineering/biotechnology. Biochem. Eng. J..

[103] Aldrich J.E. (2007). Basic physics of ultrasound imaging. Crit. Care Med..

[104] Sancheti S.V., Gogate P.R. (2017). A review of engineering aspects of intensification of chemical synthesis using ultrasound. Ultrason. Sonochem..

[105] Córdova A., Henríquez P., Nuñez H., Guerrero C., Illanes A. (2022). Recent Advances in the Application of Enzyme Processing Assisted by Ultrasound in Agri-Foods: A Review. Catalysts.

[106] Umego E., He R., Ren W. (2021). Ultrasonic-Assisted Enzymolysis: Principle and Applications. Process Biochem..

[107] Subhedar P.B., Gogate P.R. (2014). Enhancing the activity of cellulase enzyme using ultrasonic irradiations. J. Mol. Catal. B Enzym..

[108] Ma H., Huang L., Jia J. (2011). Effect of energy-gathered ultrasound on Alcalase. Ultrason. Sonochem..

[109] Ma X., Wang W., Zou M. (2015). Properties and structures of commercial polygalacturonase with ultrasound treatment: Role of ultrasound in enzyme activation. RSC Adv..

[110] Lan W., Chen S. (2020). Chemical kinetics, thermodynamics and inactivation kinetics of dextransucrase activity by ultrasound treatment. React. Kinet. Mech. Catal..

[111] Jadhav S.H., Gogate P.R. (2014). Intensification in the Activity of Lipase Enzyme Using Ultrasonic Irradiation and Stability Studies. Ind. Eng. Chem. Res..

[112] Khan A., Beg M.R., Waghmare P. (2021). Intensification of biokinetics of enzymes using ultrasound-assisted methods: A critical review. Biophys. Rev..

[113] Priya, Gogate P.R. (2021). Ultrasound-Assisted Intensification of Activity of Free and Immobilized Enzymes: A Review. Ind. Eng. Chem. Res..

[114] Wang Z., Lin X., Li P. (2012). Effects of low intensity ultrasound on cellulase pretreatment. Bioresour. Technol..

[115] De Carvalho Silvello M.A., Martínez J., Goldbeck R. (2020). Low-frequency ultrasound with short application time improves cellulase activity and reducing sugars release. Appl. Biochem. Biotech..

[116] Vartolomei A., Calinescu I., Vinatoru M., Gavrila A.I. (2022). A parameter study of ultrasound assisted enzymatic esterification. Sci. Rep..

[117] Li H., Xu M., Yao X. (2022). The promoted hydrolysis effect of cellulase with ultrasound treatment is reflected on the sonicated rather than native brown rice. Ultrason. Sonochem..

[118] De Souza Soares A., Júnior B.R.C.L., Augusto P.E.D. (2021). Ultrasound processing of amyloglucosidase: Impact on enzyme activity, stability and possible industrial applications. Acta. Sci. Technol..

[119] Sun J., Zhang Z., Xiao F. (2015). Production of xylooligosaccharides from corncobs using ultrasound-assisted enzymatic hydrolysis. Food Sci. Biotechnol..

[120] Parikh D.T., Lanjekar K.J., Rathod V.K. (2021). Ultrasound-assisted lipase catalyzed synthesis of propyl caprate: Process optimization, kinetic, and thermodynamic evaluation. Chem. Eng. Process..

[121] Hristov J. (2010). Magnetic field assisted fluidization–A unified approach. Part Mass transfer: Magnetically assisted bioprocesses. Rev. Chem. Eng..

[122] Ma H., Huang L., Zhu C. (2011). The effect of pulsed magnetic field on horseradish peroxidase. J. Food Process Eng..

[123] Portaccio M., De Luca P., Durante D. (2003). In vitro studies of the influence of ELF electromagnetic fields on the activity of soluble and insoluble peroxidase. Bioelectromagnetics.

[124] Xiong R., Zhang W., Zhang Y., Chen Y., He Y., Fan H. (2019). Remote and real time control of an FVIO–enzyme hybrid nanocatalyst using magnetic stimulation. Nanoscale.

[125] Portaccio M., De Luca P., Durante D. (2005). Modulation of the catalytic activity of free and immobilized peroxidase by extremely low frequency electromagnetic fields: Dependence on frequency. Bioelectromagnetics.

[126] Zhang J., Wang S., Xu B. (2012). Effect of alternating magnetic field treatments on enzymatic parameters of cellulase. J. Sci. Food Agr..

[127] Wasak A., Drozd R., Jankowiak D. (2019). The influence of rotating magnetic field on bio-catalytic dye degradation using the horseradish peroxidase. Biochem. Eng. J..

[128] Blanchard J.P., Blackman C.F. (1994). Clarification and application of an ion parametric resonance model for magnetic field interactions with biological systems. Bioelectromagnetics.

[129] Caliga R., Maniu C.L., Mihăşan M. (2016). ELF-EMF exposure decreases the peroxidase catalytic efficiency in vitro. Open Life Sci..

[130] Kotani M. (1968). Paramagnetic properties and electronic structure of iron in heme proteins. Adv. Quantum. Chem..

[131] Ovejero J.G., Armenia I., Serantes D. (2021). Selective Magnetic Nanoheating: Combining Iron Oxide Nanoparticles for Multi-Hot-Spot Induction and Sequential Regulation. Nano Lett..

[132] Knecht L.D., Ali N., Wei Y. (2012). Nanoparticle-mediated remote control of enzymatic activity. ACS Nano.

[133] Coffey W.T., Fannin P.C. (2002). Internal and Brownian mode-coupling effects in the theory of magnetic relaxation and ferromagnetic resonance of ferrofluids. J. Phys. Condens. Mat..

[134] Xia T.T., Lin W., Liu C.Z. (2018). Improving catalytic activity of laccase immobilized on the branched polymer chains of magnetic nanoparticles under alternating magnetic field. J. Chem. Technol. Biot..

[135] Armenia I., Bonavia M.V.G., De Matteis L. (2019). Enzyme activation by alternating magnetic field: Importance of the bioconjugation methodology. J. Colloid Interface Sci..

[136] Zheng M., Su Z., Ji X. (2013). Magnetic field intensified bi-enzyme system with in situ cofactor regeneration supported by magnetic nanoparticles. J. Biotechnol..

[137] Liu Y., Guo C., Liu C.Z. (2015). Enhancing the resolution of (R,S)-2-octanol catalyzed by magnetic cross-linked lipase aggregates using an alternating magnetic field. Chem. Eng. J..

[138] Sun J., Sun F., Xu B. (2010). The quasi-one-dimensional assembly of horseradish peroxidase molecules in presence of the alternating magnetic field. Colloid Surf. A.

[139] Xia T.T., Feng M., Liu C.L. (2021). Efficient phenol degradation by laccase immobilized on functional magnetic nanoparticles in fixed bed reactor under high-gradient magnetic field. Eng. Life Sci..

[140] Cui J., Li L., Kou L. (2018). Comparing Immobilized Cellulase Activity in a Magnetic Three-Phase Fluidized Bed Reactor under Three Types of Magnetic Field. Ind. Eng. Chem. Res..

[141] Tang W., Ma T., Zhou L. (2019). Polyamine-induced tannic acid co-deposition on magnetic nanoparticles for enzyme immobilization and efficient biodiesel production catalysed by an immobilized enzyme under an alternating magnetic field. Catal. Sci. Technol..

[142] José C., Toledo M.V., Briand L.E. (2016). Enzymatic kinetic resolution of racemic ibuprofen: Past, present and future. Crit. Rev. Biotechnol..

[143] Salgın S., Çakal M., Salgın U. (2020). Kinetic resolution of racemic naproxen methyl ester by magnetic and non-magnetic cross-linked lipase aggregates. Prep. Biochem. Biotech..

[144] Zhang Y., Wang Y., Zhou Q., Chen X., Jiao W., Li G., Peng M., Liu X., He Y., Fan H. (2021). Precise Regulation of Enzyme−Nanozyme Cascade Reaction Kinetics by Magnetic Actuation toward Efficient Tumor Therapy. ACS Appl. Mater. Interfaces.

[145] Bashari M., Jin Z.Y., Wang J.P. (2016). A novel technique to improve the biodegradation efficiency of dextranase enzyme using the synergistic effects of ultrasound combined with microwave shock. Innov. Food Sci. Emerg..

